# Postmenopausal estrogen and progestin effects on the serum proteome

**DOI:** 10.1186/gm121

**Published:** 2009-12-24

**Authors:** Sharon J Pitteri, Samir M Hanash, Aaron Aragaki, Lynn M Amon, Lin Chen, Tina Busald Buson, Sophie Paczesny, Hiroyuki Katayama, Hong Wang, Melissa M Johnson, Qing Zhang, Martin McIntosh, Pei Wang, Charles Kooperberg, Jacques E Rossouw, Rebecca D Jackson, JoAnn E Manson, Judith Hsia, Simin Liu, Lisa Martin, Ross L Prentice

**Affiliations:** 1 Public Health Sciences Division, Fred Hutchinson Cancer Research Center, 1100 Fairview Ave N, Seattle, WA 98109, USA; 2 Department of Pediatrics, University of Michigan Comprehensive Cancer Center, 1500 East Medical Center Drive, Ann Arbor, MI 48109, USA; 3 Biomarkers and Personalized Medicine Unit, Eisai Inc., 4 Corporate Drive, Andover, MA 01810, USA; 4 WHI Project Office, National Heart, Lung, and Blood Institute, National Institutes of Health, 6701 Rockledge Drive, Bethesda, MD 20892, USA; 5 Division of Endocrinology, Ohio State University, 198 McCampbell, 1581 Dodd Drive, Columbus, OH 43210, USA; 6 Division of Preventive Medicine, Brigham and Women's Hospital, Harvard Medical School, 75 Francis Street, Boston, MA 02115, USA; 7 Research and Development, AstraZeneca LP, 1971 Rockland Road, Wilmington, DE 19803, USA; 8 Division of Public Health, Epidemiology & David Geffen School of Medicine, Department of Medicine, Box 951772, Los Angeles, CA 90095, USA; 9 Department of Medicine, George Washington University, 2121 Eye St, NW; Washington, DC 20052, USA

## Abstract

**Background:**

Women's Health Initiative randomized trials of postmenopausal hormone therapy reported intervention effects on several clinical outcomes, with some important differences between estrogen alone and estrogen plus progestin. The biologic mechanisms underlying these effects, and these differences, have yet to be fully elucidated.

**Methods:**

Baseline serum samples were compared with samples drawn 1 year later for 50 women assigned to active hormone therapy in both the estrogen-plus-progestin and estrogen-alone randomized trials, by applying an in-depth proteomic discovery platform to serum pools from 10 women per pool.

**Results:**

In total, 378 proteins were quantified in two or more of the 10 pooled serum comparisons, by using strict identification criteria. Of these, 169 (44.7%) showed evidence (nominal *P *< 0.05) of change in concentration between baseline and 1 year for one or both of estrogen-plus-progestin and estrogen-alone groups. Quantitative changes were highly correlated between the two hormone-therapy preparations. A total of 98 proteins had false discovery rates < 0.05 for change with estrogen plus progestin, compared with 94 for estrogen alone. Of these, 84 had false discovery rates <0.05 for both preparations. The observed changes included multiple proteins relevant to coagulation, inflammation, immune response, metabolism, cell adhesion, growth factors, and osteogenesis. Evidence of differential changes also was noted between the hormone preparations, with the strongest evidence in growth factor and inflammation pathways.

**Conclusions:**

Serum proteomic analyses yielded a large number of proteins similarly affected by estrogen plus progestin and by estrogen alone and identified some proteins and pathways that appear to be differentially affected between the two hormone preparations; this may explain their distinct clinical effects.

## Background

Postmenopausal hormone therapy was shown to have multiple effects of public-health importance in the Women's Health Initiative (WHI) randomized, placebo-controlled hormone-therapy trials of 0.625 mg/day conjugated equine estrogen (E-alone) [1] or of this same estrogenic preparation plus 2.5 mg/day medroxyprogesterone acetate (E+P) [2], over respective average intervention periods of 7.1 and 5.6 years. The observed effects were similar for the two preparations for some outcomes, including stroke [3,4] and hip fracture [5,6]; whereas E+P effects were unfavorable (*P *< 0.05) compared with those for E-alone for other outcomes, including coronary heart disease (CHD) [7,8], breast cancer [9,10], and venous thromboembolism (VT) [11,12], and a global index [1,2] that was designed to summarize major health benefits *versus *risks [13].

Several of the articles just cited formally examined whether interactions occurred between the hormone-therapy hazard ratios and baseline study-subject characteristics. Although some moderate variations were detected (for example, for E-alone and breast cancer [10]), these tended to provide limited insight into the biologic mechanisms and pathways involved in the observed clinical effects. A cardiovascular disease nested case-control study also was conducted to relate baseline values of candidate biomarkers and post-randomization biomarker changes to observed hormone-therapy effects. This study confirmed baseline biomarker disease associations and identified some pertinent biomarker changes after hormone-therapy initiation, but identified few interactive or explanatory biomarkers for either CHD [14] or stroke [15], although the E+P hazard ratio elevation for CHD appeared to be smaller among women having relatively low baseline low-density lipoprotein cholesterol [14].

It follows that much remains to be explained about the pattern of biologic changes induced by these hormone-therapy preparations in relation to the outcome effects mentioned earlier. Proteomic discovery work has the potential to identify biomarkers that may help to explain E+P or E-alone clinical effects or differences in effects between the two preparations. Hence, we applied a comprehensive quantitative proteomic approach designated Intact Protein Analysis System (IPAS) [16-19] to compare the serum proteome at 1 year after randomization to baseline for 50 women assigned to E+P and for 50 women assigned to E-alone, in the WHI hormone-therapy trials. These women were selected to be free of major disease outcomes through the WHI clinical trial intervention phase and were selected to be adherent to their assigned hormone regimen over the first year of treatment, but were otherwise randomly selected from women assigned to active treatment in the trial cohorts. The IPAS approach involves extensive fractionation followed by tandem mass spectrometry and is capable of identifying proteins over seven orders of abundance. For reasons of throughput, serum pools were formed from 10 E+P women (five baseline and five 1-year pools), or from 10 E-alone women, before proteomic analysis.

We recently reported [20] proteomic changes from the E-alone component of this project. An impressive 10.5% of proteins had false discovery rates, for a change, of <0.05. The affected proteins had relevance to multiple pathways, including coagulation, metabolism, osteogenesis, and inflammation, among others. Ten of 14 protein changes tested were confirmed with enzyme-linked immunosorbent assays (ELISAs) in the original samples, and in serum samples from 50 nonoverlapping randomly chosen women, selected by using the same criteria, from the E-alone trial treatment group.

Here, we sought to uncover proteins and pathways that are differentially affected by E+P therapy relative to E-alone that would provide leads for the comparatively unfavorable effects with E+P observed in these trials.

## Methods

### Study cohorts

The use of human samples was approved by the Fred Hutchinson Cancer Research Center Institutional Review Board. Fifty study subjects were randomly selected from the 8,506 women assigned to active E+P in the WHI clinical trial, which also included 8,102 women assigned to placebo. All women were postmenopausal, with a uterus, and in the age range from 50 to 79 years, at recruitment during 1993 through 1998. The selected women were required to have been adherent to study medication (80% or more of pills taken) over the first year after randomization, and without a major clinical event (CHD, stroke, VT, breast or colorectal cancer, or hip fracture) over the intervention and follow-up period (through March 2005). A second nonoverlapping subset of E+P women was selected, by using the same criteria, for replication studies with ELISA. As previously reported [20], the same selection criteria were used for the E-alone discovery and replication phases of the study. Women enrolled in the E-alone trial (10,739) satisfied the same eligibility criteria as E+P enrollees, but were posthysterectomy at randomization. Women who used hormone therapy before trial enrollment had mostly stopped such treatment, months or years before enrollment, and were otherwise required to undergo a 3-month washout before randomization. Serum samples, collected at baseline and 1 year, were stored at -80°C until proteomic analyses.

### Sample preparation, protein fractionation, and mass spectrometry analysis

These methods were previously described [20] in detail and are only briefly summarized here. As in the E-alone project component, pools formed from 30 μl of serum for 10 randomly selected women from the 50 E+P group women were formed from baseline and 1-year specimens.

After immunodepletion of the six most abundant proteins (albumin, IgG, IgA, transferrin, haptoglobin, and antitrypsin), pools were concentrated, and intact proteins having cysteine residues were isotopically labeled with acrylamide (baseline pools received the 'light' C12 acrylamide; 1-year pools the 'heavy' C13 acrylamide). The baseline and 1-year pools were than mixed together for further analysis.

The combined sample was diluted, and each sample was separated into 12 subsamples by using anion exchange chromatography, and each subsample was further separated into 60 fractions by using reversed-phase chromatography, giving a total of 720 fractions for each original mixed sample. Aliquots of 200 μl from each fraction, corresponding to about 200 μg of protein, were separated for mass spectrometry 'shotgun' analysis.

Lyophilized aliquots from the 720 individual fractions were subjected to in-solution trypsin digestion, and individual digested fractions, four to 60 from each reversed-phase run, were combined into 11 pools, giving a total of 132 (12 × 11) fractions for analysis from each original mixed baseline and 1-year pool. Tryptic peptides were analyzed with an LTQ-FT mass spectrometer. Spectra were acquired in a data-dependent mode in a mass/charge range of 400 to 1,800, and the five most abundant +2 or +3 ions were selected from each spectrum for tandem mass spectrometry (MS/MS) analysis.

### Protein identification and baseline versus 1-year concentration assessment

The acquired LC-MS/MS data were automatically processed by the Computational Proteomics Analysis System [21]. Database searches were performed by using X!Tandem against the human International Protein Index (IPI) by using tryptic search. Database search results were analyzed by using PeptideProphet [22] and ProteinProphet [23].

The relative quantitation of 1-year to baseline concentration for cysteine-containing peptides identified by MS/MS was extracted by using a script designated Q3 ProteinRatioParser [16], which calculates the relative peak areas of heavy to light acrylamide-labeled peptides. Peaks with zero area were reset to a background value to avoid singularities. Peptides having PeptideProphet ≥ 0.75, Tandem expect score <0.10, and mass deviation <20 ppm were considered for quantification. Proteins were identified as those having ProteinProphet scores ≥ 0.90, and their ratios were calculated by taking the geometric mean of all the associated peptide ratios. Proteins from all 10 IPAS experiments were aligned by their protein group number, assigned by ProteinProphet, to identify master groups of indistinguishable proteins across experiments. Ratios for these protein groups were logarithmically transformed and median-centered at zero. The following protein groups were removed in this analysis: groups that had fewer than five peptide ratios across all 10 experiments; groups that contained proteins that were targeted for depletion; and groups in which all proteins had been annotated as 'defunct' by IPI.

### Statistical analysis of 1-year versus baseline protein concentrations

Protein log-(concentration) ratios were analyzed by first normalizing further, so that the median of the log-ratios is zero for all the proteins identified from a mixed baseline and 1-year sample. Concentration changes after E+P use were identified by testing the hypothesis that the mean of the log-ratios across the (up to 5) mixed samples is zero, by using a weighted moderated *t *statistic [24] implemented in the R package LIMMA [25]: the log-ratios were weighted by the number of quantified peptides for each protein, and a matrix of weights was included in the linear model. The variance was estimated by using the sum of the sample variances from the E+P and E-alone data, with the requirement of at least one degree of freedom for variance estimation. Benjamini and Hochberg's method [26] was used to accommodate multiple testing for the large number of proteins quantified, through the calculation of estimated false discovery rates (FDRs).

The same method was used to identify proteins for which the 1-year to baseline change in concentration differed between E+P and E-alone. Specifically, a moderated *t *statistic was used to test for a difference in means between the log-ratios for E+P and those for E-alone, with common log-ratio variance for the two preparations.

### Biologic pathway analysis

We developed a regularized Hotelling T^2 ^procedure (Chen LS, Prentice RL, and Wang P, submitted for publication, 2009) to identify sets of proteins, defined by biologic pathways, that change concentration with E+P, or that change differentially in the E+P and E-alone project components. This testing procedure takes advantage of the correlation structure among the log-ratios for proteins in a given set. Protein sets were defined by using the KEGG database [27,28].

To accommodate multiple hypotheses testing issues, the significance for individual proteins or for biologic pathways, is based on a 5% FDR criterion.

### ELISA-based validation

ELISAs are commercially available for some of the proteins for which evidence emerged of change after E+P use, or of differential change between E+P and E-alone. ELISA tests were applied according to manufacturer's protocols for individual baseline and 1-year serum samples from an additional randomly selected nonoverlapping 50 E+P and 50 E-alone women, for independent validation of leads from the proteomic discovery work. *P *values were obtained by applying *t *tests to log-transformed 1-year-to-baseline concentration ratios. Log-ratios from ELISA and IPAS were compared to assess discovery platform signals.

## Results

The average age at enrollment for the selected 50 E+P women is 63.2, similar to that for the trial cohort as a whole. Other study-subject characteristics were generally similar also to those for the entire trial cohort [2], as was also the case for the 50 selected E-alone women [20]. Subject characteristics for both studies are shown in Table 1. Some characteristics varied among the pools of size 10, as expected with the random assignment of women to pools. For example, the average baseline age (standard deviation) for the five E+P pools was 60.6 (8.4), 65.8 (5.3), 63.5 (8.5), 63.2 (7.1), and 62.8 (7.0), respectively. The project generated 2,576,869 spectra from the E+P pools, as compared with 2,458,506 from the E-alone pools. These led to the identification of 3,669 IPI-based proteins from the E+P pools compared with 4,679 from the E-alone analyses; and 942 IPI-based relative protein concentrations for E+P, *versus *1,054 for E-alone, including 698 that were quantified in both E+P and E-alone analyses.

**Table 1:** Baseline characteristics among women included in hormone-therapy proteomics project (n = 50 for E+P and for E-alone trials)

	E+P		E-alone		
			
	Number	%	Number	%	*P *value^a^
Age group at screening, years					0.20
50-59	17	34.0	25	50.0	
60-69	21	42.0	13	26.0	
70-79	12	24.0	12	24.0	
Minority race/ethnicity	3	6.0	8	16.0	0.20
Postmenopausal hormone therapy use					0.69
Never used	31	62.0	26	52.0	
Past user	15	30.0	19	38.0	
Current user (3-month 'wash out' before enrollment)	4	8.0	5	10.0	
Smoking					0.50
Never	34	69.4	29	58.0	
Past	14	28.6	19	38.0	
Current	1	2.0	2	4.0	
Parity					0.74
Never pregnant/no term pregnancy	6	12.0	4	8.0	
≥ 1term pregnancy	44	88.0	46	92.0	
Age at first birth, years					0.38
<20	8	21.1	15	34.1	
20-29	29	76.3	27	61.4	
30+	1	2.6	2	4.5	
Treated diabetes	2	4.0	7	14.0	0.16
Treated for hypertension or BP ≥ 140/90	15	31.9	17	37.0	0.67
History of high cholesterol requiring pills	2	4.3	2	4.5	>0.99
Statin use at baseline	0	0.0	2	4.0	0.49
Aspirin (≥ 80 mg) use at baseline	8	16.0	8	16.0	>0.99
History of MI	1	2.0	0	0.0	>0.99
History of angina	1	2.0	3	6.0	0.62
History of CABG/PTCA	1	2.0	0	0.0	>0.99
History of DVT or PE	1	2.0	0	0.0	>0.99
Family history of breast cancer (female)	6	13.0	7	14.6	>0.99
History of fracture on or after age 55	3	8.3	1	3.1	0.62
Gail Model Five Year Risk of Breast Cancer					0.26
<1	7	14.0	10	20.0	
1 - <2	31	62.0	34	68.0	
2 - <5	12	24.0	6	12.0	
Number of falls in last 12 months					0.97
None	31	66.0	30	69.8	
1 time	9	19.1	7	16.3	
2 times	6	12.8	6	14.0	
3 or more times	1	2.1	0	0.0	
					
	**Mean**	**SD**	**Mean**	**SD**	***P *value^b^**
	
Age at screening, years	63.2	7.2	61.4	7.9	0.24
Body-mass index (BMI), kg/m^2^	28.8	6.0	31.1	6.1	0.05

^a^*P *value based on Fisher's Exact test of association. ^b^*P *value based on two-sample *t *test.

MI = myocardial infarction; CABG/PCTA = coronary artery bypass graft/percutaneous transluminal angiography; DVT = deep vein thrombosis; PE = pulmonary embolus.

### Serum protein concentration ratios

Protein concentration ratios were further filtered and curated by using stringent standards (see Methods) for protein identification, including a requirement that a protein is quantified in at least two of the 10 IPAS experiments leading to a focus on 378 proteins (IPIs), all but 10 of which were quantified for both E+P and E-alone. A remarkable 169 (44.7%) of these showed evidence (nominal *P *< 0.05) of change from baseline to 1-year with E+P or E-alone, or with both. For E+P, 371 proteins were quantified under these quality standards, of which 132 (35.6%) had *P *< 0.05 as compared with 18.6 expected by chance, and 98 (26.4%) had FDRs <0.05 compared with 94 for E-alone. Of these, 84 had FDR <0.05 for both preparations. Table S1 in Additional file 1 shows estimated 1-year-to-baseline concentration log ratios for all 378 proteins ranked according to the minimum of *P *values for change with E+P or change with E-alone. Significance levels (*P *values) are also given for a test of equality of the E+P and E-alone ratios.

Table 2 lists proteins for which strong evidence (FDR < 0.01) exists of changed concentration with E+P or with E-alone, according to biologic pathways that were found to be associated with E-alone use in [20]. Nominal *P *values and FDRs for change also are provided. Of note, five proteins involved in the insulin growth factor pathway are represented in Table 2. Five of these proteins have 1.25-fold or greater changes in their concentrations with E-alone or E+P treatment or both. Protein NOV homologue (*NOV*) and insulin-like growth factor 1 (*IGF1*) were both decreased with E-alone and E+P. Insulin-like growth factor-binding protein 1 (*IGFBP1*) level was increased with both E-alone and E+P. Strong evidence of the E+P effect exists on each of blood coagulation and inflammation, metabolism, osteogenesis, complement and immune response, and cell adhesion. Moreover, the changes (base 2 logarithm of 1-year-to-baseline concentration ratio in Table 2) are mostly quantitatively very similar between E+P and E-alone, attesting to major effects of conjugated equine estrogens on the serum proteome.

**Table 2:** Gene ontology classification of proteins with statistically significant changes (FDR < 0.01) for E+P or E-alone

		E+P			E-Alone		
			
Protein	Description	Log_2 _ratio year 1 relative to baseline	*P *value	FDR	Log_2 _ratio year 1 relative to baseline	*P *value	FDR
**Blood coagulation and inflammation**							
*VTN*	Vitronectin	0.352	2.16E-07	2.68E-05	0.368	7.09E-08	8.98E-06
*CP*	Ceruloplasmin	0.679	5.19E-07	2.87E-05	0.752	3.01E-07	2.86E-05
*SERPINC1*	Antithrombin III variant	-0.196	5.05E-06	0.000157	-0.143	5.50E-05	0.00106
*PLG*	Plasminogen	0.224	1.56E-05	0.000404	0.230	1.24E-05	0.000393
*HABP2*	Uncharacterized protein	0.251	5.23E-05	0.00108	0.309	7.24E-05	0.00131
*F*12	Coagulation factor XII	0.261	0.000102	0.00158	0.252	0.000219	0.00268
*APOH*	β_2_- Glycoprotein 1	0.149	0.000199	0.00242	0.193	8.89E-05	0.00135
*ATRN*	Attractin	-0.190	0.000213	0.00242	-0.126	0.00366	0.0214
*F*9	Coagulation factor IX	0.540	0.000214	0.00242	0.572	0.000511	0.00498
*TFPI*	Tissue factor pathway inhibitor	-0.396	0.000315	0.00317	-0.369	8.49E-05	0.00135
*KNG1*	Kininogen-1	0.152	0.00106	0.00786	0.228	5.60E-05	0.00106
							
Metabolism							
*GC*	Vitamin D-binding protein	0.231	3.10E-06	0.000115	0.237	2.75E-06	0.000131
*HPX*	Hemopexin	0.123	6.65E-05	0.00118	0.117	0.000124	0.00162
*RBP4*	Plasma retinol-binding protein	0.167	0.000117	0.00161	0.177	0.000262	0.00311
*APOA2*	Apolipoprotein A-II	0.212	0.000532	0.00483	0.302	1.75E-05	0.000475
*ENPP2*	Ectonucleotide Pyrophosphatase/phosphodiesterase family member 2	0.369	0.00692	0.0333	0.650	0.000749	0.00619
							
Osteogenesis							
*FETUB*	Fetuin-B	0.783	1.09E-09	4.04E-07	0.741	1.02E-09	3.89E-07
*COL1A1*	Collagen α-1(I) chain	-0.896	5.40E-07	2.87E-05	-0.575	8.80E-05	0.00135
*AHSG*	α_2_- HS-glycoprotein	0.211	3.44E-06	0.000116	0.243	7.46E-07	5.02E-05
*NOTCH2*	Neurogenic locus notch homologue protein 2	-0.784	0.000315	0.00317	-0.062	0.648	0.815
							
Complement and immune response							
*PGLYRP2*	*N*-acetylmuramoyl-L-alanine amidase	-0.343	1.49E-06	6.17E-05	-0.335	3.72E-06	0.000141
*ORM2*	α_1_- acid glycoprotein 2	-0.181	1.63E-05	0.000404	-0.144	8.38E-05	0.00135
*C4BPA*	C4B-binding protein α chain	-0.200	3.34E-05	0.000731	-0.148	0.000377	0.00398
*CFHR1*	Complement factor H-related protein 1	0.162	8.95E-05	0.00145	0.185	2.96E-05	0.000661
*CFB*	Complement factor B	0.137	0.000106	0.00158	0.210	5.58E-06	0.000193
*C*8*A*	Complement component C8 α chain	-0.206	0.000163	0.00216	-0.202	0.000121	0.00162
*C4BPB*	C4B-binding protein β chain	-0.260	0.00018	0.0023	-0.196	0.00156	0.0112
*PGLYRP1*	Peptidoglycan recognition protein	0.321	0.000232	0.00253	-0.056	0.458	0.68
*CFHR5*	Complement factor H-related 5	0.179	0.000264	0.00281	0.241	2.76E-05	0.000656
*MASP2*	Mannan-binding lectin serine protease 2	0.200	0.000435	0.00415	0.173	0.000643	0.00568
*CFHR2*	Complement factor H-related protein 2	0.181	0.000449	0.00417	0.205	0.00016	0.00203
*C*8*B*	Complement component C8 β chain	-0.221	0.00059	0.00522	-0.199	0.0015	0.0112
*ITIH4*	Inter-α-trypsin inhibitor heavy chain H4	0.458	0.000733	0.00634	0.374	0.00495	0.0273
*VNN1*	Pantetheinase	0.477	0.000888	0.00688	0.517	0.00124	0.00963
*C*6	complement component C6	-0.123	0.00151	0.011	-0.171	0.000123	0.00162
*B*2*M*	β_2_- microglobulin	0.208	0.00205	0.0144	0.230	0.0011	0.00873
*LRG1*	Leucine-rich α_2_-glycoprotein	0.278	0.00553	0.0278	0.445	0.000582	0.0054
*MBL2*	Mannose-binding protein C	-0.190	0.00677	0.0331	-0.341	9.37E-05	0.00137
*LILRA3*	Leukocyte immunoglobulin-like receptor subfamily A member 3	-0.237	0.00874	0.0374	-0.281	0.000277	0.00319
*FN1*	Fibronectin	-0.193	0.0663	0.176	-0.358	0.000574	0.0054
							
Cell adhesion							
*ICAM1*	Intercellular adhesion molecule 1	-0.299	2.69E-05	0.000626	-0.142	0.00171	0.012
*MEGF10*	Multiple epidermal growth factor-like domains 10	-1.330	0.0365	0.114	-1.100	0.000671	0.0058
							
Growth factor activity							
*IGFBP7*	Insulin-like growth factor-binding protein 7	-0.295	0.000404	0.00395	-0.133	0.0342	0.109
*NOV*	Protein NOV homologue	-0.759	0.00083	0.00672	-0.344	0.0123	0.0506
*IGF1*	Insulin-like growth factor IA	-0.353	0.000981	0.00745	-0.371	0.000403	0.00414
*IGFBP1*	Insulin-like growth factor-binding protein 1	0.528	0.00242	0.0158	1.270	3.66E-06	0.000141
*IGFBP4*	Insulin-like growth factor-binding protein 4	0.179	0.102	0.234	0.511	0.000697	0.00588
							
Other							
*SHBG*	Sex hormone-binding globulin	1.460	1.14E-08	2.13E-06	1.450	2.57E-08	4.88E-06
*AGT*	Angiotensinogen	1.150	3.09E-07	2.87E-05	1.200	2.05E-06	0.000111
*LUM*	Lumican	-0.382	4.81E-07	2.87E-05	-0.163	0.00175	0.0121
*TFF3*	Trefoil factor 3	2.590	8.84E-07	4.11E-05	2.160	2.06E-05	0.000522
*OAF*	Out at first protein homologue	0.398	7.32E-06	0.000209	0.393	4.17E-05	0.000881
*A1BG*	α_1B_-Glycoprotein	0.167	5.71E-05	0.00112	0.266	7.92E-07	5.02E-05
*ABI3BP*	Target of NESH-SH3	-0.290	6.07E-05	0.00113	-0.184	0.00149	0.0112
*CLEC3B*	Tetranectin	-0.240	7.65E-05	0.00129	-0.158	0.00102	0.00827
*FBN1*	Fibrillin-1	-0.365	0.000114	0.00161	-0.269	1.45E-05	0.000423
*DBH*	Dopamine β-hydroxylase	-0.262	0.000207	0.00242	-0.186	0.00203	0.013
*FGA*	Fibrinogen α chain	0.364	0.00076	0.00643	0.303	0.00741	0.0373
*SPARCL1*	SPARC-like protein 1	-0.311	0.0008	0.00661	-0.043	0.508	0.722
*CPN2*	Carboxypeptidase N subunit 2	0.161	0.000865	0.00685	0.190	0.000331	0.0036
*AMBP*	AMBP protein	0.116	0.00283	0.0174	0.148	0.00061	0.00552
*PEAR1*	Platelet endothelial aggregation receptor 1	-0.650	0.00948	0.0379	-0.722	0.000424	0.00424
*AFM*	Afamin	0.058	0.119	0.259	0.177	0.00033	0.0036

*P *value = significance level for test of no change in protein concentration; FDR = estimated false discovery rate for this test.

Table 3 presents the differences in quantitative ratios for E+P minus E-alone that were nominally significantly different (*P *< 0.05) from each other. For this analysis, we tested the 368 proteins meeting our identification and quantification criteria that were common to both E+P and E-alone. Twenty-six proteins were identified with nominal *P *values <0.05 for differential change between E+P and E-alone. The list includes proteins involved in insulin growth factor binding and inflammation. Insulin growth factor-binding proteins (IGFBPs) may be affected differently by E+P compared with E-alone. Specifically, three proteins show nominally statistically significant differences with E+P compared with E-alone. *IGFBP1 *is increased (log_2 _ratio, 1.27) with E-alone, but the increase is mitigated with E+P (log_2 _ratio, 0.528). *IGFBP4 *is increased with E-alone (log_2 _ratio, 0.511), but not with E+P (log ratio, 0.179). *NOV*, also known as *IGFBP9*, is decreased with E-alone (log_2 _ratio, -0.344) and decreased to a greater extent with E+P (log_2 _ratio, -0.759).

**Table 3:** Difference in Year 1 from baseline concentration ratios (E+P minus E-Alone) for all proteins with difference of *P *< 0.05

Protein	Description	Difference of log_2 _ratios (year 1 relative to baseline): E+P minus E-alone	P-Diff	FDR
*LUM*	Lumican	-0.219	0.00141	0.317
*IGFBP1*	Insulin-like growth factor-binding protein 1	-0.742	0.00227	0.317
*PGLYRP1*	Peptidoglycan recognition protein	0.377	0.00259	0.317
*NOTCH2*	Neurogenic locus notch homologue protein 2	-0.723	0.00426	0.33
*ACTB*	Actin cytoplasmic 1	0.667	0.00527	0.33
*LYVE1*	Lymphatic vessel endothelial hyaluronic acid receptor 1	-0.217	0.00538	0.33
*AZGP1*	α_2_-Glycoprotein 1 zinc	0.496	0.00776	0.408
*ICAM1*	Intercellular adhesion molecule 1	-0.158	0.0126	0.511
*SPARCL1*	SPARC-like protein 1	-0.268	0.0138	0.511
*COL1A1*	Collagen α-1(I) chain	-0.321	0.015	0.511
*A1BG*	Á_1B_-glycoprotein	-0.099	0.0153	0.511
*SEPP1*	Selenoprotein P	0.756	0.0181	0.549
*F*5	Coagulation factor V	0.301	0.0194	0.549
*CFL1*	Cofilin-1	0.597	0.023	0.549
*C6orf115*	Similar to protein C6ORF115	1.070	0.0233	0.549
*MMP2*	72-kDa type IV collagenase	1.190	0.0246	0.549
*HRG*	Histidine-rich glycoprotein	-0.136	0.0282	0.549
*ABCA9*	ATP-binding cassette subfamily A member 9	3.280	0.0284	0.549
*MMRN1*	Multimerin-1	0.691	0.0293	0.549
*AFM*	Afamin	-0.120	0.0298	0.549
*MCAM*	Cell-surface glycoprotein MUC18	-0.357	0.0409	0.614
*NOV*	Protein NOV Homologue	-0.415	0.0409	0.614
*TFF2*	Trefoil factor 2	-1.920	0.0412	0.614
*ECM1*	Extracellular matrix protein 1	-0.184	0.0412	0.614
*IGFBP4*	Insulin-like growth factor-binding protein 4	-0.332	0.0442	0.614
*CFB*	Complement factor B	-0.073	0.0444	0.614

P-Diff = significance level for test of equality of protein-concentration ratios for E+P and E-alone; FDR = estimated false discovery rate for this test.

### Protein-set analyses

In addition to the protein classifications presented earlier, the 368 proteins quantified for both E+P and E-alone were subjected to protein set analysis. In total, 41 KEGG human disease pathways were represented by at least two proteins in this group. Each protein has been quantified in at least two IPAS experiments in both E+P and E-alone. Table 4 indicates pathways that show a baseline *versus *1-year difference for E+P and E-Alone at FDR < 0.05 by using a regularized Hotelling *T*^2 ^test. We also tested the equality of log-concentration ratios between the two regimens for proteins in these pathways by using the same test statistic. Two pathways had FDR < 0.05 (Table 5). The gonadotropin-releasing hormone (GnRH) signaling pathway, known to be regulated by estrogen, was represented by two proteins (matrix metalloproteinase 2 (*MMP2*) and phospholipase A_2 _(*PLA2G1B*)), and a pathway associated with bladder cancer was represented by three proteins (*MMP2*, thrombospondin 1 (*THBS1*), and vascular endothelial growth factor C (*VEGFC*)). Both pathways had nominal *P *values of 0.002, with corresponding FDRs of 0.041. *MMP2*, a collagenase with the ability to break down extracellular matrix proteins, was common to both pathways, and substantially explains the difference between the two regimens for these pathways.

**Table 4:** KEGG pathways having two or more quantitated proteins for which evidence of differential change between baseline to 1-year concentration with E+P and E-alone was significant, with FDR < 0.05

E+P Pathway	Number quantified protein	*P *value^a^	FDR
Porphyrin and chlorophyll metabolism	2	<0.001	<0.001
Dorsoventral axis formation	4	<0.001	<0.001
Motor signaling pathway	2	0.002	0.009
Pancreatic cancer	2	0.002	0.009
Focal adhesion	14	0.003	0.011
Bladder cancer	3	0.005	0.015
Renal cell carcinoma	3	0.009	0.023
Notch signaling pathway	5	0.011	0.024
Ether lipid metabolism	2	0.012	0.024
Long term depression	2	0.022	0.039
Regulation of actin cytoskeleton	8	0.025	0.041
Cytokine-cytokine receptor interaction	21	0.032	0.048
			
E-alone pathway			

Porphyrin and chlorophyll metabolism	2	<0.001	<0.001
GNRH signaling pathway	2	<0.001	<0.001
Ether lipid metabolism	2	0.001	0.011
Bladder cancer	3	0.002	0.016

^a^From a regularized Hotelling *T*^2 ^test.

**Table 5:** KEGG pathways having two or more quantitated proteins for which evidence of differential change between E+P and E-alone was significant, with FDR < 0.05

E+P *versus *E-Alone	GNRH signaling pathway	Bladder cancer
Number of proteins	2	3
Proteins in the pathway	*MMP2*, *PLA2G1B*	*MMP2*, *THBS1*, *VEGFC*
*P *value^a^	0.002	0.002
FDR^a^	0.041	0.041

^a^From a regularized Hotelling *T*^2 ^test.

### ELISA-based protein assays in an independent set of subjects

**Figure 1:**
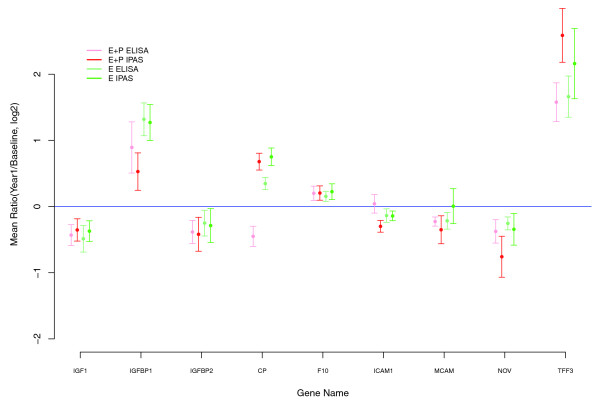
Mean log_2_-transformed ratios (95% confidence interval): Intact Protein Analysis System and enzyme-linked immunosorbent assay (ELISA).

The 26 proteins with nominal *P *< 0.05 for differential change between E+P and E-alone based on IPAS mass spectrometry findings each had FDR > 0.3, so that many of these may be attributable to chance. We sought to determine whether concordant changes in these proteins can be demonstrated in an independent set of subjects and with independent methods. Figure 1 shows 1-year-to-baseline concentration log-ratios (95% confidence intervals (CIs)) from IPAS mass spectrometry data along with corresponding values from ELISA evaluation of 1-year-to-baseline ratios for an independent set of 50 women selected from the active-treatment group in the E+P trial. Corresponding IPAS and ELISA information also is provided for E-alone. We observed concordance of IPAS and ELISA data between the two sets of subjects for six of eight proteins assayed. The lack of replication for ceruloplasmin (*CP*) and *ICAM1 *may be due to multiple comparison effects in the discovery component or other factors, notably distinct epitope targets by ELISA assays compared with quantified peptides by mass spectrometry.

## Discussion

These analyses show that 1 year of use of E+P has a profound effect on the serum proteome, with more than a fourth (26.4%) of quantified proteins having FDR < 0.05 for change. Eight proteins with altered levels were further tested in an independent set of samples. ELISA assays of six of the eight proteins showed changes concordant with the mass spectrometry data. The correlation of initial concentration ratios by mass spectrometry with ELISA ratios from an independent set of samples supports the reliability of the protein changes observed. Our previous report on E-alone [20] provided a detailed discussion of the proteins that changed after treatment with conjugated equine estrogens, in which 19% of proteins were changed after 1 year of treatment. Findings for 10 proteins were confirmed and validated by ELISA assays. Proteins altered with E-alone therapy had relevance to processes such as coagulation, inflammation, growth factors, osteogenesis, metabolism, and cell adhesion, among others. The most striking feature of the present E+P analysis is the similarity in these quantitative proteomic changes when medroxyprogesterone acetate is added to the daily conjugated equine estrogen. For changes with E+P, 98 proteins had FDR < 0.05 compared with 94 proteins for E-alone. Of these, 84 proteins had FDR < 0.05 for both preparations, and corresponding intensity ratios tended to be quite similar between the two regimens for most of these proteins. Hence, our prior discussion [20] of proteins and pathways that were changed after E-alone is largely applicable to the E+P hormone preparation as well. The 1 year of aging between the baseline and 1-year blood-sample collection could have some influence on the serum proteome, but any such influence should be absent for the comparison of E+P *versus *E-alone changes, because age-related changes would apply equally for the two regimens.

**Figure 2:**
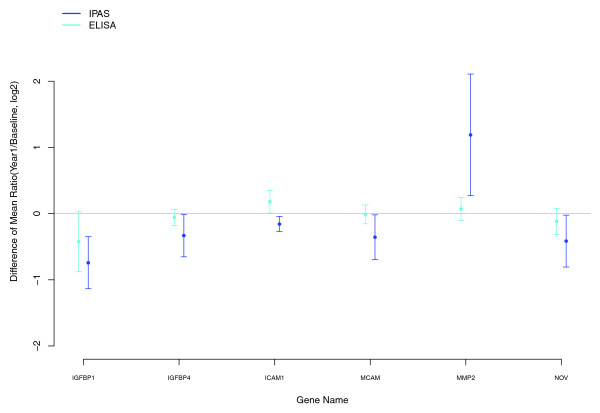
Differences of mean log_2_-transformed ratios (95% CI): Intact Protein Analysis System and enzyme-linked immunosorbent assay (ELISA).

When we specifically sought proteins for which the change with E+P differed from that for E-alone, a number of potential proteins (Table 3) emerged, but chance could not be ruled out as an explanation for any particular protein. To check whether these suggested differences could be attributable to differences in the E+P and E-alone study cohorts (Table 1) we repeated the Table 3 analyses with the mean age and mean BMI at baseline in each pool as adjustment factors. The log intensity ratio differences were not appreciably affected by this adjustment, although *P *values tended to become less significant because of reduction in 'degrees of freedom' for the moderated *t *tests. Interestingly, after this adjustment, the FDR for *NOTCH2 *decreased to 0.02. None of the other FDRs in this sensitivity analysis were < 0.05. Aberrant NOTCH signaling has been implicated in tumorigenesis and has been reported to play an oncogenic role in breast cancer [29,30]. A decrease of *NOTCH2 *serum levels with E+P, but not E-alone, could be related to alteration of signaling and increased risk of breast cancer with E+P but not with E-alone. A differential change between E+P and E-alone in *IGFBP1 *was supported by ELISA data in an independent set of 50 subjects for each regimen (Figure 2) and may provide an important lead to understanding clinical effects that differ between the two preparations, including breast cancer. As elaborated later, Table 3 also contains proteins that are associated with atherogenesis.

First, consider proteins involved in the insulin growth factor-signaling pathway. The overall pattern (Table 3) is a greater increase in insulin growth factor-binding proteins (*IGFBP1*, *IGFBP4*) with E alone compared with E+P, whereas the decrease in *NOV *was relatively greater with E+P. ELISA testing produced trends in these same directions for all three proteins, but only that for *IGFBP1 *approached statistical significance in the independent set. Collectively, these analyses suggest that progestin may attenuate some of the estrogen-induced increases in IGF-binding proteins.

It has been previously suggested that medroxyprogesterone acetate has only a weak degree of opposition to the estrogen-induced decrease of total *IGF-1 *(which is primarily of hepatic origin), in agreement with our study findings for *IGF-1 *levels [31]. However, given reduced levels of IGF-binding proteins, it would be expected that less IGF is bound, possibly increasing the availability of free IGF. The IGF-signaling pathway plays a role in cell proliferation, tissue development, and tumorigenesis. The IGF pathway has been linked to colorectal malignancy [32,33], and serum levels of *IGF1 *have been associated with colon cancer risk [34]. Changes in the IGF pathway with E+P compared with E-alone could potentially explain some of the differences in the clinical outcomes. In particular, *IGF-1 *is a strong mitogen, and varying levels of free *IGF-1 *between E+P and E-alone treatment could help explain the increased risk of breast cancer with E+P.

In addition to proteins related to the IGF pathway, several other proteins of biologic interest in the context of E+P *versus *E-alone effects on the serum proteome are presented in Table 3. Expression of the α_2_-glycoprotein 1 zinc (*AZGP1*) gene is regulated predominantly androgens and progestins [35,36]. Our data suggest an increase in *AZGP1 *protein levels with E+P and a decrease with E-alone. *AZGP1 *has been identified as a potential prognostic marker for early-stage breast cancer and a useful immunohistochemical marker of apocrine cell differentiation in human breast tissue [37]. Increased levels of circulating *AZGP1 *in E+P compared with E-alone may be associated with increased risk of breast cancer in the former group. Circulating levels of extracellular matrix proteins (collagen α-1 chain (*COL1A1*), lumican (*LUM*), and extracellular matrix protein 1 (*ECM1*) may also be differentially affected by E+P compared with E-alone, whereas *MMP2*, a metalloproteinase that breaks down *COL1A1*, may be increased with E+P compared with E. The extracellular matrix plays a variety of physiological roles, many of which are related to cancer, including tumor invasion. Changes in the extracellular matrix with E+P compared with E-alone could also help explain the differences in cancer risks associated with these treatments.

Several proteins listed in Table 3 have been linked to atherogenesis, and thus may suggest avenues for exploring mechanisms underlying a more-substantial early increase in CHD risk with E+P than with E-alone in the randomized trials. For example, matrix metalloproteinases (for example, *MMP2*) are thought to participate in atherogenic inflammation [38]. The role of innate immunity in atherogenesis is less well established; *PGLYRP1 *participates in recognition of bacteria by neutrophils, but is independently associated with coronary artery calcification and abdominal aortic plaque [39]. The difference in *PGLYRP1 *concentration among women taking E+P *versus *E-alone suggests a possible mechanistic link.

Other extracellular matrix proteins (Table 3) may also shed light on the relation between E+P and CHD events. One such protein, lumican (*LUM*), contributes to variation in proteoglycan composition of arterial intima by location within the human vasculature. Enhanced deposition of lumican has been observed in the intima of the atherosclerosis-prone internal carotid artery compared with the internal thoracic artery, a relatively atherosclerosis-resistant vessel [40]. The relation between serum and tissue proteoglycan levels, the impact of proteoglycan composition on plaque stability, and the clinical significance of lumican all remain to be determined.

Our proteomic comparisons (Table 3) may also provide insight into the greater elevation in venous thromboembolic event risk when progestin was added to conjugated estrogens. For example, coagulation factor V (*F5*) binds to multimerin 1 (*MMFN1*), with high affinity for storage in human platelet granules, and may modulate thrombosis [41].

Some important considerations exist in assessing the effects of estrogens and progestins broadly on the serum proteome. The preparations considered here are conjugated equine estrogen and medroxyprogesterone acetate. Further studies would be needed to determine whether the changes reported here also arise for the other estrogen (for example, 17β-estradiol) and progesterone (norethisterone acetate or levonorgestrel) treatments. Related to this, these substances are taken orally, and the first-pass hepatic metabolism of oral estrogens is known to stimulate a wide variety of proteins, synthesized in the liver. Of the 378 proteins reported here, 73 are included in the liver-specific gene set listed in Hsiao and colleagues [42] (Table S1 in Additional file 1). For example, of 66 significantly upregulated proteins (FDR < 0.05), 35 are in the liver-specific gene path, as were 28 of 71 for E-alone.

Of the proteins emphasized in the preceding discussion, *IGFBP1*, *AZGP1*, and *F5*, but not others, are part of the liver-specific list. Given that transdermal estrogen, which is being increasingly used in clinical practice to treat menopausal symptoms, bypasses the liver, these proteins may not be affected when estrogen is administered transdermally.

## Conclusions

In summary, E+P, like E-alone, has a profound effect on the serum proteome and affects multiple pathways that are relevant to observed clinical effects on cancer, cardiovascular disease, and fractures, among others. The addition of 2.5 mg/d medroxyprogesterone acetate to 0.625 mg/d conjugated equine estrogen may have an impact on the IGF pathway proteins and may affect circulating levels of extracellular matrix proteins (for example, *MMP2*) of potential relevance to the less-favorable E+P effects, compared with those for E-alone, on breast cancer, and CHD. Similarly the addition of medroxyprogesterone acetate may also augment the effects of conjugated estrogens on coagulation factors (for example, factor V), of potential relevance to a relatively greater elevation in venous thromboembolism with E+P. These and other leads from our proteomic study will benefit from further testing in women who experienced major clinical outcomes and in matched controls from the WHI hormone therapy trials, to evaluate more directly the potential of these protein-concentration changes to contribute to a biologic explanation for observed trial-outcome patterns.

## Abbreviations

*AZGP1*: α_2_-glycoprotein 1 zinc; CHD: coronary heart disease; CIs: confidence intervals; *COL1A1*: collagen α-1 chain; *CP*: ceruloplasmin; E: conjugated equine estrogen; E+P: conjugated equine estrogen plus medoxyprogesterone acetate; *ECM1*: extracellular matrix protein 1; ELISA: enzyme-linked immunosorbent assay; *F5*: coagulation factor V; FDR: false discovery rate; GnRH: gonadotropin-releasing hormone; *ICAM1*: intercellular adhesion molecule 1; IGF: insulin-like growth factor; IGFBP: insulin-like growth factor-binding protein; IPAS: Intact Protein Analysis System; IPI: International Protein Index; LC-MS/MS: liquid chromatography tandem mass spectrometry; *LUM*: lumican; *MMFN1*: multimerin 1; *MMP2*: matrix metalloproteinase 2; *NOV*: protein NOV homologue; *PGLYRP1*: peptidoglycan recognition protein; *PLA2G1B*: phospholipase A_2_; *THBS1*: thrombospondin 1; *THY1*: THY-1 membrane glycoprotein; *VCAM1*: vascular cell adhesion protein 1; *VEGFC*: vascular endothelial growth factor C; VT: venous thromboembolism; WHI: Women's Health Initiative.

## Competing interests

The authors declare that they have no competing interests.

## Authors' contributions

SJP, SMH, CK, JR, RDJ, JEM, JH, SL, LM, and RLP participated in drafting the manuscript. Data acquisition was performed by HW and HK. Data were analyzed and interpreted by SJP, SMH, LA, LC, SP, HK, QZ, MM, PW, and RLP. Immunoassays were performed by TBB and MMJ. SMH and RLP were responsible for the study design. Statistical analysis was performed by AA, LC, MM, PW, and RLP.

## Additional files

The following additional files for this article are available online: Additional file 1 contains Table S1, which shows year 1 to baseline log-transformed concentration ratios after estrogen plus progestin (E+P) or estrogen (E-Alone) exposure for all 378 quantified proteins.

## Supplementary Material

Additional file 1 Table S1 shows year 1 to baseline log-transformed concentration ratios after estrogen plus progestin (E+P) or estrogen (E-Alone) exposure for all 378 quantified proteins. Click here for file
